# Advances in the Synthesis and Application of Anti-Fouling Membranes Using Two-Dimensional Nanomaterials

**DOI:** 10.3390/membranes11080605

**Published:** 2021-08-09

**Authors:** Asif Shahzad, Jae-Min Oh, Mudassar Azam, Jibran Iqbal, Sabir Hussain, Waheed Miran, Kashif Rasool

**Affiliations:** 1 Department of Energy and Materials Engineering, Dongguk University-Seoul, Seoul 04620, Korea; asifshzd8@gmail.com (A.S.); jaemin.oh@dongguk.edu (J.-M.O.); 2 Institute of Chemical Engineering & Technology, University of Punjab, Lahore 54590, Pakistan; mudassar.azam@tuwien.ac.at; 3 College of Natural and Health Sciences, Zayed University, Abu Dhabi 144534, United Arab Emirates; Jibran.Iqbal@zu.ac.ae; 4 Department of Environmental Sciences and Engineering, Government College University, Faisalabad 38000, Pakistan; HucainSabir@gmail.com; 5 International Center for Materials Nanoarchitectonics, National Institute for Materials Science, Tsukuba 305-0044, Japan; 6 Qatar Environment and Energy Research Institute, Hamad Bin Khalifa University (HBKU), Qatar Foundation, Doha 5824, Qatar

**Keywords:** anti-fouling, membranes, two-dimensional, nanomaterials, MXenes, graphene

## Abstract

This article provides a comprehensive review of the recent progress in the application of advanced two-dimensional nanomaterials (2DNMs) in membranes fabrication and application for water purification. The membranes fouling, its types, and anti-fouling mechanisms of different 2DNMs containing membrane systems are also discussed. The developments in membrane synthesis and modification using 2DNMs, especially graphene and graphene family materials, carbon nanotubes (CNTs), MXenes, and others are critically reviewed. Further, the application potential of next-generation 2DNMs-based membranes in water/wastewater treatment systems is surveyed. Finally, the current problems and future opportunities of applying 2DNMs for anti-fouling membranes are also debated.

## 1. Introduction

Water scarcity has become an increasingly complex challenge with the growth of the global population, economic expansion, and climate change, highlighting the demand for advanced water treatment technologies that can provide clean water in a scalable, consistent, cheap, and sustainable manner. As water scarcity continues to be a global challenge, there is a need to explore various pathways to close the water consumption cycle at the nexus of food, water, and energy systems [1]. There is a paradigm shift needed towards the circular economy considering municipal and industrial wastewater as a valuable source of clean water, nutrients, and fertilizer instead of a grave environmental issue [2]. In fact, treated water has been considered as an alternative source to augment the clean water supply and tackle issues caused by a shortage of fresh water in different parts of the world, especially arid regions [1]. Recent interest in the reuse of wastewater has spurred new research aiming to convert wastewater treatment plants into resource recovery facilities [3]. Resource recovery and the reuse of wastewater is an important and viable option for water-scarce regions. This can be achieved using chemo-physical and/or biological processes to minimize the concentration of pollutants in water to acceptable levels [4,5,6,7,8]. Several approaches including adsorption, precipitation, coagulation/flocculation, electrochemical methods, ion exchange, and membrane filtration have been studied over the years to decontaminate industrial and domestic wastewater [9]. However, membrane filtration is considered to be one of the best practical approaches towards water treatment [10].

The membrane provides a physical barrier for contaminants and is one of the key methods for water purification. The use of microfiltration, ultrafiltration, nanofiltration, and reverse osmosis (RO) membranes, depending on the pore size and molecular size, is rapidly increasing for removal of different organic and inorganic pollutants from various kinds of effluent streams [11,12,13].

Furthermore, forward osmosis, membrane distillation, and electrodialysis can also address the need for efficient desalination solutions [14]. Membrane technology is rapidly developing and growing largely based on the realization of its potential in almost every industrial sector including the water purification industry [5,15]. Polymeric membranes are being employed in water/wastewater purification and desalination owing to their outstanding permeability, mechanical characteristics, and relative cost-effectiveness [16,17]. Fouling is one of the greatest bottlenecks of membrane technology for purification of the effluents generated from different industrial processes in addition to moderate permeate flux, and high thermal energy requirements [18]. Nevertheless, there are still substantial efforts required to further progress on finding the anti-fouling and thermally resistant new materials for polymeric membranes fabrication. Recent research is focused on the fabrication of mixed-matrix membranes (MMMs), hydrophilic modification, and crosslinking of membrane materials to achieve the above-mentioned goals [19].

**Figure 1:**
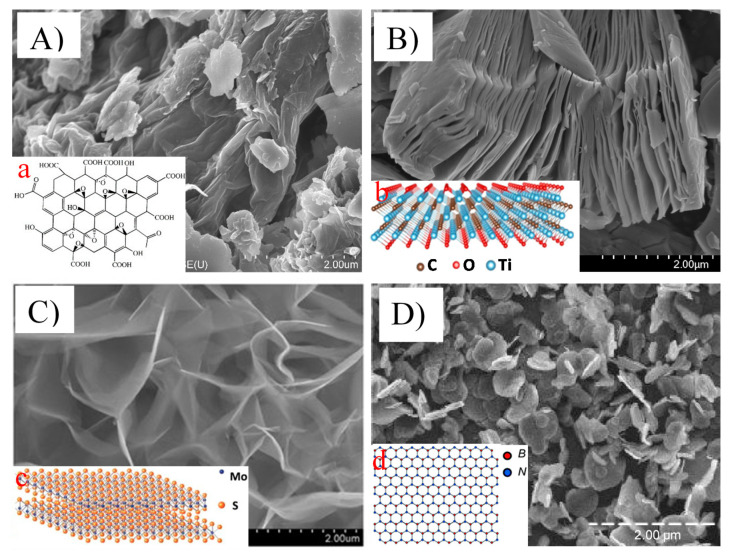
Scanning electron microscopic images showing the morphology of ((**A**) and (**a**)) graphene oxide nanosheets (reproduced from [23,24] with permission from the Royal Society of Chemistry and Dove Medical Press Limited, respectively), ((**B**) and (**b**)) titanium carbide (Ti_3_C_2_) MXene nanosheets (reproduced from [25,26] with permission from the Elsevier), ((**C**) and (**c**)) MoS_2_ nanosheets (reproduced from [22,27] with permission from the Royal Society of Chemistry and Elsevier, respectively), and ((**D**) and (**d**)) boron nitride nanosheets (reproduced from [27,28] with permission from the MDPI and Royal Society of Chemistry, respectively).

Novel advanced nanomaterials are being developed and used for surface functionalization, as adsorption materials, nanocatalysts, coatings, and membranes fabrication [6,20]. Recent advancements in two-dimensional nanomaterials (2DNMs) open a new pathway for addressing the grand challenge of water treatment owing to their unique structures and superior properties. Emerging 2D nanostructures (Figure 1) such as graphene, MoS_2_, MXenes, boron nitride (h-BN), g-C_3_N_4_, and black phosphorus have demonstrated an unprecedented surface-to-volume ratio, which promises ultralow material use, ultrafast processing time, and ultrahigh treatment efficiency for water cleaning [21,22].

In this review, we provide a state-of-the-art account of engineered 2DNMs in the synthesis of different types of membranes and their application for emerging water/wastewater purification technologies. Starting with the type of membrane fouling, the fundamental design strategies of 2DNMs are discussed with an emphasis on their physicochemical properties, underlying mechanism, and targeted applications in different scenarios. This review aims to guide the design of next-generation 2DNMs for the development of selective, multifunctional, programmable membranes for different types of water effluent treatment. A perspective on the pressing challenges and emerging opportunities in 2DNMs-enabled membranes synthesis and application for wastewater treatment is also presented.

## 2. Membrane Fouling Types and Antifouling Strategies

**Figure 2:**
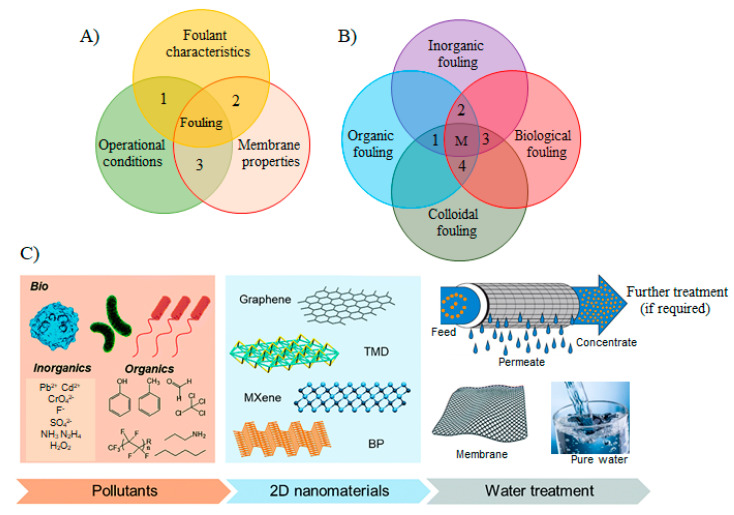
(**A**) Factors affecting the membrane fouling: (1) foulant characteristics such as concentration, size, solubility, diffusivity, hydrophobicity, charge, etc.; (2) membrane properties such as hydrophobicity, surface roughness, pore size, charge on membranes surface, and surface functional groups; (3) operational conditions such as flux, medium temperature, and flow velocity, medium chemistry, pH, ionic strength, and presence of organic/inorganic matters (reproduced from [31] with permission from the Elsevier. (**B**) A schematic graphic of the various fouling mechanisms corresponding to the fouling materials. In the real membrane processes, fouling occurs as mixed fouling, i.e., the combination of different fouling mechanisms happening concurrently. The dotted lines in the diagram with areas 1, 2, 3, 4 and M show the different examples of mixed fouling between two or more fouling mechanisms. (**C**) A schematic illustration of the water/wastewater treatment processes utilizing the versatile behavior of 2DNMs in efficient membrane separation (reproduced from [39] with permission from the Royal Society of Chemistry).

The fouling of membranes is normally described as the undesirable accumulation of organic compounds, inorganic salts, colloids, and microorganisms on/in the membranes surface/interior in the course of filtration [29]. Fouling mainly damages the performance of membranes by affecting permeate output, increases transmembrane pressure, and shortens the effective operation time [30]. Given that fouling is quite a complicated process that is influenced by various mechanisms in its formation on/in the membrane surface, they should be grasped to have a better strategy to minimize, mitigate, and clean the fouling. The major aspects that affect fouling can be categorized into four groups [31] (Figure 2A): (1) foulant characteristics such as charge, molecular size, concentration, diffusivity, solubility, polar nature, etc.; (2) membrane properties such as surface roughness, hydrophobicity, pore size, surface charge, and functional groups of surface; (3) operational conditions such as flux, temperature, and flow velocity, solution chemistry, pH, ionic strength, and the presence of organic/inorganic matters. While the types of fouling that will occur on the membrane surface is largely influenced by the types, concentrations, and properties of foulants appear in the influent water and its solution chemistry, interactions between the foulant and the membrane surfaces could increase the fouling propensity; therefore, membrane properties can largely affect the fouling. On the other hand, operational conditions such as the temperature and pressure of influents and flow velocity can also influence the degree of fouling.

Major foulants responsible for fouling can be categorized into inorganic, organic, colloids, and/or biological (Figure 2B). Inorganic fouling is usually initiated with the deposition of inorganic precipitates such as metal hydroxides, and scales on the membrane’s surface or membrane pores [32]. Scaling typically denotes to the formation of deposits of inverse-solubility salts such as calcium carbonate and calcium phosphate that is very concerning for RO and NF owing to their rejection to inorganic species that build a concentration layer close to membrane surface and facilitates precipitation. On the other hand, the organic fouling is triggered by the buildup of organic materials from the process streams, such as macromolecules including proteins, carbohydrates, and antifoams [17,32]. Proteins have one of the most complex fouling features because of their labile and dynamic nature [33]. For instance, proteins in the process stream fold together after interacting with each other and form aggregates which subsequently concentrate near the membrane and precipitate on the membrane’s surface, leading to grave membrane fouling. Colloidal fouling in the membrane systems is generally caused by the convective depositions of colloids (clays, silica, paint pigments, etc.) on the membrane surface. The colloidal fouling tends to create additional layers (cake layer) on the membrane surface [34]. Microbial/biological fouling is also a very important type of fouling, which facilitates in developing the undesirable microbial layers on membrane surfaces in the form of biofilm and can be also found in harsh conditions and environments [35,36]. Bacteria in the water systems is considered a promoter of biofouling in membrane processes. Bacterial cells after growth also produce by-products and soluble bacterial products, some of which are high-molecular-weight compounds composed mostly of proteins, lipids, and polysaccharide constituents. Furthermore, biofilms also participate in enhancing inorganic precipitation via enhanced nucleation, and crystallization kinetics [16,37]. Nevertheless, mostly, there are no single fouling mechanism proceeds in real membrane processes, but a combination of various fouling materials and mechanisms take place that makes it more problematical to deal with as depicted in Figure 2B.

To overcome these challenges triggered by fouling, numerous antifouling approaches have been developed. In antifouling approaches, physical disruption of biofilm matrices, control by biocides (e.g., quorum quenching and phage-based decomposition), and chemically modifying the anti-fouling behavior of membranes (coating with nanoparticles, etc.) are prominent approaches so far [36]. The approaches to modify antifouling membranes are generally classified as either passive or active [36]. Passive approaches count on modifying membrane surfaces to impede foulant adhesion and facilitate removal. For instance, fouling resistance can be increased by modifying the membrane surface to make it more hydrophilic. Furthermore, fouling release involves the formation of amphiphilic surfaces, which helps in driving the foulants away from the membranes. However, while hydrophilicity in the membrane surface can effectively resist the onset of fouling by non-migratory foulants (e.g., biomacromolecules and normal organic matter), they often fail to markedly resist fouling from spreadable foulants, particularly oils [38]. Thus, fouling cannot always be suppressed by membrane hydrophilization. Therefore, the formulation of antifouling membranes with wide-ranging applicability is greatly desirable. In comparison, active antifouling approaches have a focus on removing the foulants by destroying them via membrane surface contact or the release of various agents. Since one antifouling mechanism and approach can help deal with a limited range of foulants only, incorporating multi-antifouling mechanisms such as passive–passive, active–active, or/and passive–active, and antifouling membranes with novel modification approaches to instill robust antifouling properties are highly important for opening many avenues for future sustainable water purification (Figure 2C). To this end, variety of 2DNMs have been designed and developed such as graphene, graphitic carbon nitride (g-C3N4), MXenes, transition metal dichalcogenides (TMDs), transition metal oxides (TMOs), metal organic frameworks (MOFs), covalent organic frameworks (COFs), many other 2D nanostructures through different methods for dealing with these foulants to achieve water purification.

## 3. Synthesis of 2DNMs Membranes

**Figure 3:**
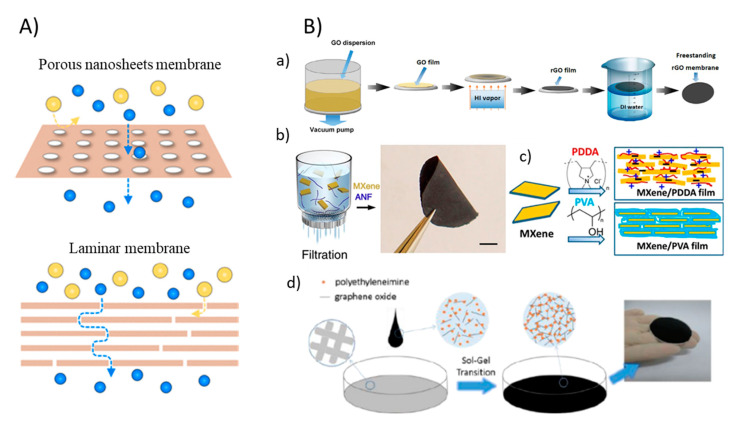
(**A**) Graphic of two types of 2DNMs membrane: porous nanosheet membranes and laminar membranes (reproduced from [41] with permission from the American Chemical Society). (**B**) Fabrication of 2DNM membranes: (**a**) A schematic illustration of freestanding rGO membranes fabricated by vacuum-assisted method (reproduced from [44] with permission from the Wiley Online). (**b**) Synthesis of free-standing MXene/ANF (reproduced from [45] with permission from the nature) and (**c**) MXene/polymer membrane by vacuum-assisted filtration methods (reproduced from [46] with permission from the PNAS). (**d**) Schematic illustration of the formation of an NLGM via the sol-gel process (reproduced from [48] with permission from the royal society of chemistry) [44].

The diverse properties of 2DNMs offer vast opportunities for the development of 2DNMs-based membranes that can possess a large density of nanochannels spread across the material grain, high flux, exceptional salt rejection, and persistent antifouling capability [40,41]. The ultrathin nanosheets with integrated structures are necessary for manufacturing excellent performing 2DNM membranes. Generally, a top-down approach, which involves exfoliating the bulk layered crystals, and bottom-up approach, which involves direct production from the prime building units, are utilized to manufacture ultrathin 2DNM nanosheets [41]. Subsequently, the 2DNM nanosheets can be fabricated into continuous separation layers by using different approaches such as filtration, spin coating, hot dropping, etc. Given nanosheets are usually either porous or nonporous, resultant membranes are classified into two types: a porous nanosheet membrane and a laminar membrane (Figure 3A). While porous nanosheet membranes are comprised of single or a few layers of 2D materials with built-in or drilled in-plane nanopores that allows permeation of molecules or ions selectively, the laminar membranes are manufactured by compiling nanosheets into laminates having interlayer channels for the transport of molecules. Selection of materials such as the membrane base material, additives, solvent, and 2DNMs depends on the intended application of the membranes. Several approaches have been employed to introduce 2DNMs to membranes, such as spray coating, spin coating, layer-by-layer assembly, Langmuir–Blodgett assembly, inkjet printing, vacuum filtration, and self-assembly at the interface of two phases [42].

While 2DNMs can be fused into/onto a membrane by making a blend of 2DNMs and membrane fabricating materials or by appending them to membranes surface by physical or chemical methodologies, properties such as high hydrophilicity, antimicrobial, flexible functionality, strong hydration capability can be achieved which provides extraordinary opportunities in mitigating membrane fouling [43]. To this end, phase inversion and electrospinning methods, involving inter-facial polymerization, graft polymerization, and dip coating as membrane modification processes are the major methods reported in membrane synthesis. The vacuum-assisted filtration process of membrane fabrication is also considered as a simple and effective method. This filtration method is not only simple and highly reproducible but also permits the very good control for thickness of the 2DNM layers. For instance, Zhang et al. used a facile and efficient approach to manufacture freestanding ultrathin rGO membranes by using filtration methods [44]. Further hydriodic acid (HI) vapor was used for the reduction of GO membrane into rGO membrane (Figure 3B(a)). Two-dimensional MXene-based composite membrane can also be synthesized with a vacuum-assisted process. Feng et al. reported MXene/Kevlar nanofiber composite membranes fabricated by filtration of MXene/ANF mixture on the anodized aluminum filter film [45]. Once dried in air for 24 h, the MXene/ANF composite membranes could simply be peeled from the substrate (Figure 3B(b)). In another work, Ti_3_C_2_T_x_ MXene and polymer composite membranes were synthesized by vacuum filtration (Figure 3B(c)) [46]. In another approach termed the sol-gel process, a non-laminated GO membrane crosslinked by polyethyleneimine was fabricated (Figure 3B(d)). In the as-prepared membranes, the GO nanosheet remains disordered as in the sol states to develop a random GO self-assembled structures that led to much larger flux in comparison to general laminated GO membrane fabricated via vacuum filtration or spin coating due to the lower flow resistance [47]. Furthermore, a brief overview about different methods applied for 2DNMs-based membrane fabrication aimed at incorporating antifouling properties are given in Table 1.

## 4. DNMs-Based Membrane for Water/Wastewater Treatment

### 4.1. Oily Produced Water

In the oil industry, produced water generally refers to the water that is generated as a byproduct while extracting oil and natural gas. Produced water is the prevalent waste stream generated in oil and gas industries with an estimated global production of 41 million m^3^ day^−1^, which is largely mixed with the dispersed oil, greases, dissolved, and suspended solids [58]. Discharging of produced water pollutes surface and underground water as well as soil and hence represents a significant issue of environmental concern. The treatment of produced water involved physical, chemical, and biological methods, and since offshore platforms have space constraints, compact physical and chemical systems are preferred [58]. However, conventional technologies are considered to be less efficient as they are either susceptible to fouling or are insufficient to separate the stabilized oil-water emulsions. Therefore, it is in great demand to develop effective techniques to treat produced water in order to satisfy the stringent governmental limitations and preserve the environment. Based on the major contaminants present in the produced water, treatment goal includes de-oiling, desalination, degassing, suspended solids removal, organic compounds removal, heavy metal and radionuclides removal, and disinfection. These treatment goals are essentially the same for potable, non-potable reuse, or disposal, although the level of contaminant removal required for potable reuse can be significantly higher, depending on the quality of the produced water. Furthermore, achieving the various treatment goals requires the use of multiple treatment technologies, and membrane processes in combination with other physical, chemical, and/or biological treatment processes helped achieve the desired goal [59]. To this end, 2DNMs-based membranes have been successfully developed for oil-water separation in water treatment [60]. For instance, GO and reduced GO (rGO) nanocomposite membranes have been developed using common blade coating and phase inversion techniques for the treatment of produced water from the oil and gas industry [61]. GO was incorporated into polybenzimidazole (PBI) matrix and only a few weight percentages of GO resulted in much enhanced oil-removal efficiency up to 99.9%, while biofouling investigation of the nanocomposite membrane over six months showed a significant improvement in comparison to pristine PBI membrane [62]. The incorporation of GO into the polymeric matrix not only improves the transport of water but also enhances the membrane’s ability to endure high operating pressures owing to the oxygenated functional groups, spacing between inter-layers, and atom-thin structures.

Since produced water also contains high levels of salts, ions, and metals such as high concentration of CaCO_3_ and different cations at near saturation level, any membrane process including membrane distillation (MD) is prone to foul rapidly. To overcome this problem, CNT immobilized membranes (CNIM) have been reported to show relatively lower fouling. Humoud et al., reported that the CNIM demonstrated greater water vapor flux and antifouling characteristics in comparison to the pristine membrane. There was a decline in 18.2% normalized flux with the polytetrafluoroethylene (PTFE) membrane after 7 h of operation compared to the CNIM [63]. Furthermore, salt deposition on the membrane surface was 77% less in the CNIM. The presence of CNTs on the membrane surface also facilitated the regenerability of the membrane and results showed regaining of 90.9% of its initial water flux after washing with CNIM. Additionally, nanofiber membranes (NFMs) have been employed for produced water treatment as it offers favorable properties including large surface area, huge porosity, tunable wettability, larger permeability, and smaller basis weight. Especially membrane surface modification is employed for reducing the fouling and the promotion of crosslinking of nanofibers to improve the mechanical strength of the membrane. For example, Ao et al. successfully fabricated the super hydrophilic GO@electrospun CNF membrane, which demonstrated a high separation efficiency, excellent antifouling properties, as well as a high flux for the gravity-driven oil/water separation [64].

### 4.2. Oily/Petroleum Wastewater

Oil pollution, which badly affects human health and other living organisms, is a serious global issue because of the large amounts of oily wastewater produced [65]. Oily wastewater sources are very wide-ranging, as the oil in the petroleum industry, petroleum refining, oil storage, transportation, and petrochemical industries in the production process generate large volumes of oily wastewater [66]. To date, different methods have been used for oil–water separation, such as coagulation/flocculation, gravity separation, skimming, and flotation [67,68]. Although these methods are found to be effective for the removal of free oil, with oil droplet size >150 μm, they are relatively inefficient for the separation of dispersed oil in the range of 20–150 μm. The use of 2DNMs in membrane technology has attracted significant attention due to the tunability of these materials, making it possible to filter impurities previously thought not possible, such as oil separation. To this end, graphene oxide (GO), carbon nanotubes (CNTs), and carbon nanofibers (CNF) have been used for the fabrication of nanocomposite membranes for oil-water separation [69].

Inspired by the two-dimensional structure and hydrophobic property, some researchers have begun to use graphene or graphene-related materials to fabricate super hydrophobic materials for oil–water separation in recent years [70]. However, these membranes tend not to be efficient because the higher density of water can hinder oil permeation by forming a layer that acts as a barrier on the membrane surface [65]. Therefore, methods to increase the hydrophilicity have been developed such as using carboxyl, hydroxyl and amine modified graphene attached polyacrylonitrile-co-maleimide (G-PANCMI) on polyethersulfone (PES) hollow fiber membrane [71] or coating the membranes with a mussel-inspired polydopamine (PDA) layer to enhance the oil/water separation via its antifouling properties and strong resistance to the adhesion of microorganisms [71]. Additionally, novel hierarchically structured membranes have been developed by assembling GO sheets on the surface of electrospun aminated polyacrylonitrile (APAN) fibers. These were super hydrophilic and had low oil-adhesion, exhibited ultra-high flux (10,000 L m^−2^ h^−1^), preferable rejection (≥98%), and remarkable antifouling performance for the separation of oil-water emulsion [71].

In other 2DNMs, the beneficial advantages of adding CNTs to membranes include increased hydrophilicity and oleophobicity [71] and improved thermal [72] and mechanical properties of the composite membranes. Consequently, CNTs-integrated composite membranes with polyvinyl alcohol (PVA) barrier layers have been developed for the treatment of oil-containing wastewater by separating oil from water [73]. In other CNTs-based membrane modifications, a hybrid system involving a photocatalytic reactor and a membrane permeation cell was utilized. In this hybrid system, polyvinylidene fluoride (PVDF)/multi-walled carbon nanotube (MWCNTs) nanocomposite membranes were fabricated to enhance the rejection, flux, and fouling resistance for full filtration of pollutants from photocatalytic reactors such as decomposed refinery wastewater and TiO_2_ photocatalyst [73]. In addition, PSf/Pebax composite membrane were developed by coating with different loadings of functionalized (F)-MWCNTs wherein the water contact angle was decreased by increasing F-MWCNTs loading which led to increased membrane hydrophilicity. Tensile strength and thermal stability of the membrane were increased by addition of F-MWCNTs. Furthermore, permeate flux was increased with increasing F-MWCNTs content up to 0.5 wt%, but permeate flux decreases with further increasing F-MWCNTs from 0.5 wt% to 2 wt% [73]. A novel Ag/polyacrylic acid (PAA)–CNTs hybrid membranes for the treatment of oil/water/solid three-phase system has also been developed. These membranes effectively separate a wide range of surfactant–stabilized oil-in-water emulsions with a remarkable flux of 3000 L m^−2^ h^−1^ bar^−1^.

CNFs are another type of carbon material that has shown high oil sorption capacity for oil-water separation. Although CNFs and CNTs have similar mechanical strength and associated properties, CNFs hold a much larger functionalized surface area compared with CNTs [69]. CNFs based membranes have been developed with many modifications to achieve effective oil-water separation. For instance, the main restriction of using CNFs for oil-water separation, i.e., low rigidity of macrostructure of CNFs can be resolved by adding silica (SiO_2_) into membrane structure [74]. Electrospun nanofibrous CNFs have also been developed which provides a quick and easier separation of oil and hence makes these membranes potential candidates for industrial applications [75]. Although carbon-based membranes can achieve very high performance, they are not able to be mass produced due to their relatively high cost. Further optimization is required for the commercialization of carbon-based membranes for oily water/wastewater.

In the last few years, a group of 2DNMs have been augmented by a large family of transition metal carbides, nitrides and carbonitrides generally known as MXenes. Titanium carbide (Ti_3_C_2_T*_x_*) was the first MXene synthesized and extensively studied for water/wastewater treatment and other environmental remediation applications owing to its unique physic-chemical properties such as higher hydrophilicity, surface area and surface functionality [21]. To date, different type of MXenes have been developed and used for the synthesis of membranes for its application in water/wastewater treatment including oil water separation [75,76,77,78]. Recently, a hydrophilic membrane was synthesized by simple coating of 2D Ti_3_C_2_T*_x_* MXene nanosheets on commercial print paper as the substrate and applied for the oil and water separation [79]. The fabricated membrane showed over 99% oil/water separation efficiency while maintaining a water flux of 450 L m^−2^ h^−^ bar^−^ with excellent anti-fouling properties.

### 4.3. Seawater and Brackish Water Desalination

The main dissimilarity between the seawater and brackish water is the amounts of dissolved salts/solids. While seawater comprised of higher levels of dissolved salts/solids ranging from 15,000 mg/L to over 40,000 mg/L, brackish water contains only 1000–15,000 mg/L of dissolved salts/solids. Since power generation required to treat the water is proportional to the salt contents of the water, the higher pressure/electric power is required to treat seawater in comparison to brackish water using membranes [80]. Therefore, energy requirements and costs for seawater desalination through membranes is much higher than brackish water. Additionally, the developed 2DNMs such as graphene which are employed solely as NF membranes were unable to reduce the seawater salinity to drinking water standards but help in treating mildly brackish feed waters only. Hence, for seawater desalination, owing to much higher salts concentration, a significant modification in membranes is required along with using RO membranes instead of NF [9].

Nevertheless, seawater desalination based on membranes is one of the most energy-efficient approaches presently employed. Researchers are largely interested in 2DNMs for seawater treatment since ion-sieving either via in-plane pores or channels of stacked nanosheets can be achieved and both of which can be economically produced at industrial-scales [81]. The fabrication of ultrathin membranes with excellent mass transport and high-water flux is possible due to hyper-thinness of 2D monolayers [82,83]. In addition, 2DNMs can significantly enhance the solute selectivity due to their structural and chemical homogeneity [84].

**Figure 4:**
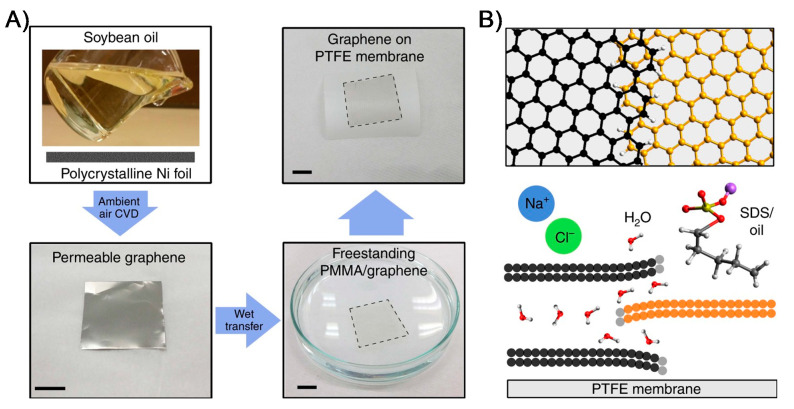
Synthesis of graphene film for water desalination membranes. The schematic (**A**) depicts the formation of permeable graphene using polycrystalline Ni substrate through ambient-air CVD process using soybean oil as a renewable source. The synthesized permeable graphene film was transferred to commercial PTFE-based MD membrane for water desalination testing. (**B**) The proposed mechanism of water purification and desalination is enabled by unique graphene features such as overlapping of graphene domains and grain boundaries as an advantageous feature in forming antifouling, long-term flux stable MD membrane (reproduced from [85] with permission from the springer nature).

In 2DNMs, graphene-based materials exhibit a great potential to be used as membranes for desalination. Water desalination via a membrane distillation process have been demonstrated using a graphene membrane where water permeation is enabled by nanochannels of multilayer, mismatched, partially overlapping graphene grains (Figure 4) [85]. Importantly, in this work, Seo et al. [85], used soybean oil as the renewable source for synthesizing graphene film by a single-step ambient-air CVD process. Graphene film was later used as an active layer in MD and demonstrated the higher rates of water vapor flux, exceptional salt rejection, stability in the longer term and sustained antifouling. Furthermore, a real-world application of membrane was validated by treating seawater from Sydney harbor for 72 h with macroscale membrane size of 4 cm^2^, while ~0.5 L per day was processed. Single-layer porous graphene has been used as a desalination membrane wherein nanometer-sized pores were developed in a graphene monolayer using an oxygen plasma etching process. This allows the size of the pores to be tuned, and as a result, the membrane exhibits a salt rejection rate of nearly 100% and rapid water transport [86]. While monolayer graphene is strong, the formation of defects and nanosized pores could deteriorate its mechanical properties for membrane applications [82]. This anomaly can be solved by utilizing an interconnected matrix of nanotubes [87]. Yang et al. grew defect-free graphene followed by incorporation with single-walled carbon nanotube (SWCNTs) backbone, simultaneously solving the issues faced by defects, pore sizes, pore chemistry and mechanical stability. The other alternative to the 2D nanoporous monolayer membranes is the 2D stacked membranes where the solute passes via nanochannels between nanosheets. Stacked membranes benefit from having channels with uniform size and smaller size distribution, parameters that are difficult to control in nanoporous thin membranes [88].

CNTs also have great potential for membrane desalination (MD) given their high aspect ratio, large surface area, high mechanical strength, and chemical robustness. In general, CNTs can be applied either as direct filters or as a filler to enhance desalination performance [89]. Membrane distillation simulations have been used to explore the water-membrane interactive nature and the dynamics through CNTs for forward osmosis applications. It was inferred that the highest water flux can be achieved with CNTs of a specific diameter (i.e., 1.08 nm or 10.8 Å) while maintaining 100% salt rejection [90]. The functionalizing of CNTs such as covalent modification or fluorination led to higher dispersibility and interfacial reactions of CNTs in the polymer, which ultimately developed the super-hydrophobic nature of the membrane. This process can be through surface fluoro-salinization, covalent functionalization, followed by the electro-spinning of the nanofibers. The water flux value obtained by this system was 48.1 L m^−2^ h^−1^ [91]. Additionally, immobilization of CNTs on the polytetrafluoroethylene (PTFE) membrane enhances the water flux rate up to 69 L m^−2^ h^−1^, for a salt concentration of 34,000 mg L^−1^ at 70 °C in a direct contact MD mode [92]. In one of the studies for using MWCNTs in desalination, chlorine-resistant reverse osmosis membranes were manufactured using MWCNTs/aromatic polyamides (PA) in which MWCNTs changes the surface roughness of the membrane but the use of chlorine for the prevention of biofouling causes membrane degradation. However, MWCNTs above 12.5% show degradation resistance against chlorine exposure while maintaining efficiency, suggesting the development of carbon nanotube/polymer composite membrane in RO water desalination technology [92].

MXenes are also promising aspirants for water desalination owing to their hydrophilicity, high surface area, and excellent mechanical and electrical properties. Smaller ions from water can be eliminated due to constrained interlayer spacing of MXenes. The water flux through MXene (Ti_3_C_2_T_x_)-based membranes was high, and the membranes exhibited excellent separation potential for various ions based on charge and hydration radius of the ion [78]. To this end, self-cross-linked MXene membrane through a facile thermal treatment has been synthesized. Self-crosslinking reaction (−OH+ −OH = − O− + H_2_O) led to the formation of Ti–O–Ti bonds between the MXene nanosheets and the resulting membrane exhibited excellent stability and anti-swelling properties in addition to the improved performance of the ion exclusion (98.6% of NaCl) compared to pristine MXene [57]. MXene-derived membranes supported on α-Al_2_O_3_ have been synthesized, which were adjusted by regulating the sintering temperature. The water and salt permeation were greater for membrane calcined at 200 °C [93].

### 4.4. Toxic Metal Wastewater

Metal-contaminated wastewater is the major source of pollution in aqueous environments; metals, particularly heavy metals, are persistent in aquatic systems and do not tend to degrade naturally [94]. Heavy metals including As, Cr, Cd, Pb, Hg, Cu, and Zn are the most prevalent pollutants in water systems [21], which are being discharged directly or indirectly into the environment by our industries and other anthropogenic activities [23]. Hasty industrial progress such as insecticides and fertilizer industries, battery and energy storage industries, electroplating (metal plating), and to some extent, paper industries are the foremost producers of toxic metals containing wastewater [95]. The heavy metals are non-degradable, carcinogenic, and have the tendency to accumulate in living systems [96]. Even at trace levels, heavy metals are toxic and cause harm to humans as well as other living organisms. In humans, heavy metals can cause short- and long-term illnesses such as headaches, nausea, depression, kidney and liver damage, and cancer. Therefore, it is of great importance to decontaminate heavy-metal-contaminated wastewater before being released into the environment.

**Figure 5:**
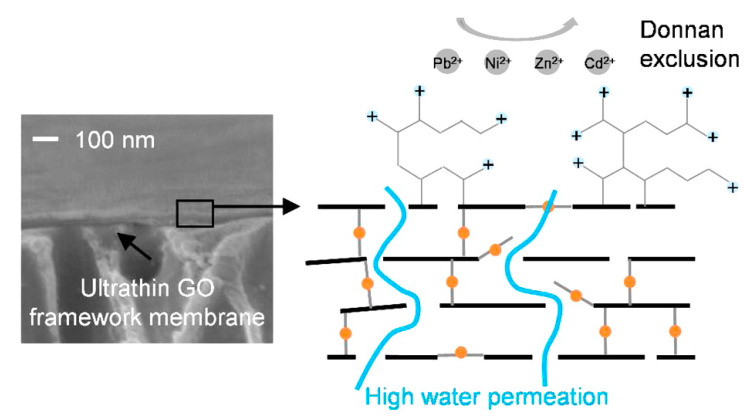
An illustration of heavy metal rejection in graphene oxide framework membrane (reproduced from [110] with permission from the American Chemical Society).

Several approaches have been developed and used over the years to remove heavy metals from aquatic systems, most of which include ion exchange, solvent extraction, chemical precipitation, flocculation and coagulation, alum and iron coagulation, sulfide precipitation, and membrane filtration (Table 2) [96,97,98,99,100]. However, most of these technologies produced a large amount of secondary waste, which further require treatment and consequently resulted in high operating costs. Moreover, low removal capacities, high start-up time, and limited removal selectivity are the foremost disadvantages of these technologies [101,102,103,104,105]. Among these treatment methods, membrane filtration stands out because it provides comparatively higher efficiencies, low cost, and selectivity [106,107]. Membrane-based desalination techniques are currently considered more environmentally friendly and energy-efficient than thermal desalination methods such as multistage flash and multiple-effect distillation [108]. Although the traditional membrane-based filtration technique is the most efficient technique to date, it suffers from low treatment capacity and high capital costs. Moreover, the conventional membranes are prone to fouling due to the presence of organic matter in the water, suffer from flux decline under high pressure, undergo rapid degradation, and have low tolerance to high temperature, acids/alkaline, and chlorine [23]. Hence, there is a pressing need for developing novel membranes with high water permeability coupled with high salt rejection capacity and better anti-fouling features which can reduce the energy consumption of the water treatment process. The ideal membrane should provide higher flux and selectivity, improved stability, and resistance to fouling. Additionally, it should be as thin as possible and mechanically robust to maximize permeability, should be chemically inert, and must retain a high salt rejection rate throughout its service life. 2DNMs such as GO can be well-dispersed in water because of the electrostatic repulsion between the ionized functional group in GO nanosheet (Table 3) [63]. Wei et al. examined the fast permeation of water through graphene oxide; water molecules pass through the nano-pores between the edge of GO sheets and between the interlayer spacing of the GO layer (Figure 5) [91]. In another study, Chang et al. reported cross-linking and construction of a GO framework by ethylenediamine (EDA), amine-enrichment modification by hyperbranched polyethyleneimine (HPEI). This kind of crosslinking provides stable and highly charged GO framework membranes with the GO selective layer thickness of 70 nm for effective heavy metal removal [109]. The crosslinked GO membrane demonstrated a superior water permeability of 5.01 L m^−2^ h^−1^ bar^−1^ and high rejections for Mg^2+^, Pb^2+^, Ni^2+^, Cd^2+^, and Zn^2+^. The application of graphene oxide-based membrane in water purification including heavy metal removal is highly promising [47,92].

### 4.5. Organic Contaminants Removal

**Figure 6:**
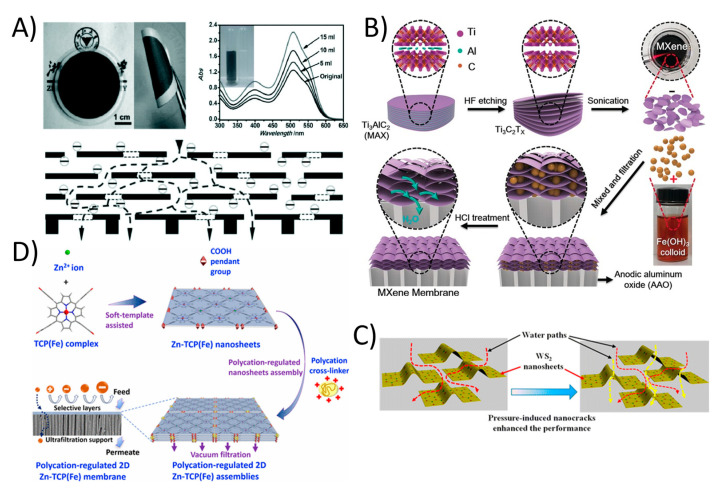
(**A**) Ultrathin (≈22–53 nm thick) graphene nanofiltration membrane (uGNM) coated on an AAO substrate with schematic illustration of the possible permeation route (reproduced from [115] with permission from the Wiley Online). (**B**) MXene membrane preparation (reproduced from [116] with permission from the Wiley Online). (**C**) The assembly of chemically exfoliated WS2 nanosheets act as an ultrafast separation membrane for small molecules with size of about 3 nm (reproduced from [117] with permission from the American Chemical Society) and (**D**) 2D MOF based defect-free ultra-thin 2DNMs membrane was fabricated by assembly of 2D Zn-TCP (Fe) nanosheets (reproduced from [118] with permission from the American Chemical Society).

The occurrence of organic contaminants (OCs) including pharmaceutical compounds (PhACs), pesticides, and endocrine disrupting chemicals (EDCs) in domestic and industrial sewages has recently become a major environmental health concern [112]. The presence of OCs in water bodies has an effect on environmental toxicity that affects microorganisms. While most of these contaminants are being regulated and new guidelines are in progress for protecting water quality emphasizes the growing demand to monitor and decrease some PhACs in wastewater treatment plants (WWTPs), where they are typically only partially eliminated [112]. There are several methods in which WWTPs can control the conversion and removal of these compounds, including biotransformation, adsorption onto biosolids and enhanced oxidation processes [113]. Most of these traditional technologies are unable to complete eliminate these persistent organic condiments. However, membrane filtration, especially 2DNMs using membranes, has shown great potential in this regard. 2DNMs including GO and derivatives, MXenes, zeolites, metal organic frameworks (MOFs), transition metal dichalcogenides (TMDCs), and covalent organic frameworks (COFs) have been utilized to fabricate 2DNMs membranes for organic contaminants at trace level. 2DNMs have shown excellent capability in organic solvent nanofiltration, which represents an important separation process in the organic phase [114]. For example, the uGNM (Ultrathin, ≈22–53 nm thick graphene nanofiltration membrane) can effectively remove organic dyes such methyl blue and direct red from water with higher rejection rate (>99% with pure water flux 21.8 L m^−2^ h^−1^ bar^−1^); see Figure 6A [115]. Furthermore, controlling the space between the graphene sheets and chemical modification can increase the antifouling properties of GO. In another study related to 2D MXene, Min et al. reported titanium carbide (Ti_3_C_2_T*_x_*) MXene with mixed cellulose ester Ti_3_C_2_T*_x_*/MCE membrane. The Ti_3_C_2_T*_x_*/MCE composite membrane possessed characteristics of both MXenes with ultrathin interlayer distance of laminate and MCE layer with high porosity, which demonstrate high water permeability and exceptionally reject methylene blue dye Figure 6B [116]. The Ti_3_C_2_T*_x_*/MCE composite membrane showed 119.83 ± 6.22 L m^−2^ h^−1^ pure water flux with a 100% rejection rate for methylene blue. The water permeance through the MXene membrane was much higher than that of the most membranes with similar rejections. Long-time operation also reveals the outstanding stability of the MXene membrane for water purification. In another study, the chemically exfoliated 2D tungsten disulfide (WS_2_) nanosheets were assembled into a layered membrane and used to selectively separate small molecules from water; see Figure 6C. The 300 nm thick WS_2_ membrane was able to reject > 90% of Evans Blue (EB; molecular size: ~3 nm) at water flux of up to 730 L m^−2^ h^−1^ bar^−1^ [117]. Furthermore, a defect-free ultra-thin 2D MOF membrane was also fabricated by assembly of 2D Zn-TCP (Fe) nanosheets [118]. The 48 nm thick 2D MOF membrane showed an exceptionally high water flux of 4243 L m^−2^ h^−1^ bar^−1^ and more than 90% of methyl red was rejected (Figure 6D). 2D material-based membranes are promising material system for promising organic removal applications. Indeed, 2DNMs signify a distinctive option for the production of membranes with nano-channels for selective transportation of ions/organic molecules [119].

### 4.6. Anaerobic/Aerobic Membrane Bioreactors for Biological Treatment

Industrial wastewater treatment is frequently problematic primarily because of rapid variations in their composition including changes in high chemical oxygen demand (COD), pH, salinity, etc. and the existence of synthetic or natural elements that inhibit or are poisonous for the activated sludge microorganisms [120]. The occurrence of the substances can inhibit biological activity which effects the system removal performance. Both anaerobic/aerobic membrane bioreactors (MBRs) treatment technology have been effectively deployed in the treatment of a variety of industrial and processing effluents. In MBRs technology, wastewater remediation is accomplished via a combination of biological unit and membrane filtration unit [114]. MBRs can remove toxic pollutants of emerging concern from wastewater at high efficiencies. However, the main shortcoming of this technology is membrane biofouling.

**Figure 7:**
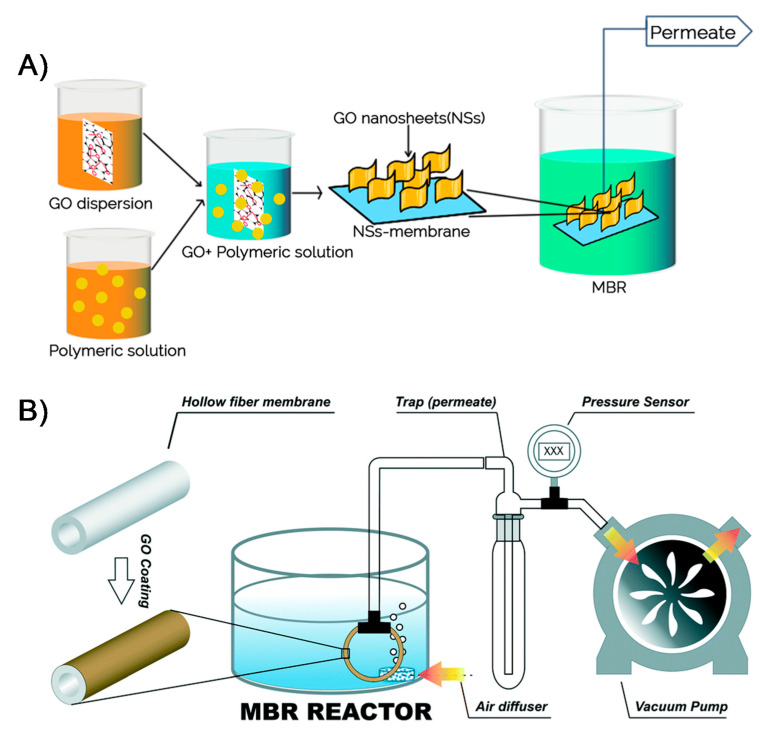
(**A**) A graphical illustration of GO-based membrane fabrication and use in MBR operation (reproduced from [114] with permission from the Springer Nature), and (**B**) schematic illustration of lab scale MBR (reproduced from [126] with permission from the Royal Society of Chemistry).

A number of efforts have been exerted to improve the performance and overcome membrane biofouling of MBRs [121,122]. The use of nanomaterials, particularly 2DNMs, for membranes has been proposed as an effective antifouling strategy with enhanced removal performance. Different kinds of 2DNMs, including 2D boron nitride membranes [28], 2D titania membranes [123], 2D graphitic carbon nitride membranes [124], molybdenum disulfide membranes, and GO membranes [125], have been tested for water purification. Amongst 2DNMs, the GO-based membranes have showed superior efficiency Figure 7A. For the MBR system, Fathizadeh et al. reported novel polyether sulfone (PES) hollow fiber membranes coated with single-layer GO (SLGO) and effectively examined these membranes in MBR [126]. The roughness of pure PES membrane surface (44 nm) was decreased (33 nm) after loading of SLGO, owing to the UV-irradiation method (Figure 7B). In comparison with pure PES membrane, UV-treated GO/PES hollow fiber membranes demonstrated exceptional permeability (65 L m^−2^ h^−1^ bar^−1^) with the minimal fouling (<15% permeance decline). The excellent performance could be due to the grafting of additional functional groups, for instance, carboxyl or hydroxyl groups for the duration of UV-irradiation onto GO flakes, which boosted the performance.

**Figure 8:**
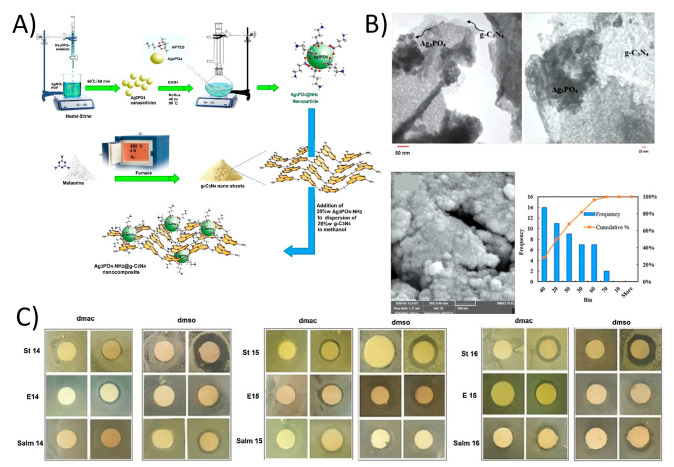
(**A**) The schematic of synthesis of Ag_3_PO_4_-NH_2_/g-C_3_N_4_ nanocomposite and (**B**) TEM and FESEM images of Ag_3_PO_4_-NH_2_/g-C_3_N_4_ along with nanoparticles size distribution chart. (**C**) The disc diffusion results of composite membrane against *Escherichia* *coli*, *Staphylococcus* and *Salmonella* (reproduced from [127] with permission from the Elsevier).

Ghalamchi et al. prepared a Ag_3_PO_4_-NH_2_/g-C_3_N_4_ nanocomposite containing PES microfiltration membrane (Figure 8A,B) [126]. Membrane was fabricated by using a phase inversion process. The addition of 2D g-C_3_N_4_ developed the hydrophilicity of membrane compared to the pure PES membrane. Therefore, Water flux was boosted from 262 L m^−2^ h^−1^ to 360 L m^−2^ h^−1^ with a loading of just 0.5 wt.% Ag_3_PO_4_-NH_2_/g-C_3_N_4_ nanocomposite on the PES membrane surface. Additionally, the antifouling capability was enhanced with an FRR of 57.5% and this was higher than pure PES membrane (39.1%) (Figure 8C).

## 5. Prospectus and Challenges

2DNMs-based membrane systems for various applications as demonstrated in many studies evidently prove the potential of this material. Since the new 2DNMs-based membrane designs have provided great benefit for developing more efficient, functional, stable, anti-fouling membranes, more specific separation objectives can be achieved. However, scaling up and manufacturing 2DNMs-based membrane systems in a cost-effective manner which meets industrial standard remains the major challenge. Exploration of exfoliation methods that can fabricate the layered materials on a large scale is required for mass production of 2DNMs and ultimately applications to membranes development. Additionally, although excellent separation functioning of 2DNMs-based membranes has been demonstrated, their durability in the long-term separation processes has yet to be confirmed. Therefore, durability-associated studies should be executed for a better understanding of the long-term durability. Given current investigations of the separation performance of 2DNMs are generally performed under comparatively ideal or less severe conditions, evaluations under practical environments can raise additional challenges for 2-DNMs and certainly help in bringing them closer to commercial applications. More insight into the processes taking place at the fluid-2DNMs’ membrane interface and the best controlling strategies of these processes are crucial for further advancing the membrane efficiency. Further increasing the membrane stability in aqueous environment and enabling high flux cross-membrane ion transport without any cross-linkers could be a game changer. In this regard, new 2DNMs with robust and desirable interlayer van der Waals interaction and weak electrostatic repulsion without any modification are vital for further progressing the field. Since 2DNMs with atomic scale thickness can assist as new building blocks for the fabrication of ultrathin membranes which can contribute to the fast and selective transportation of small molecules/ions, molecular transport pathways manipulation by improving the behavior of 2D nanosheets assemblies, alteration of the microstructure of interlayer channels, and physicochemical properties manipulation is required to meet the requirements in 2DNMs membranes. Furthermore, research for improving the 2DNMs membranes with anti-swelling characteristics having interlayer sizes below hydration radius of cations is important for eliminating the cumbersome interlayer tuning process, which could make the fabrication process significantly simpler and boost the stability of membranes, especially desalination membranes. While many synthesizing techniques involved toxic chemicals, greener and safer synthesis methodologies for 2DNMs need to be established to satisfy sustainable production demands.

Given 2-DNMs have pros and cons, the selection of one carbon nanomaterial over the other can be based on improved functional properties, effectiveness, and environmental aspects. For instance, CNTs-based membranes usually provide the large permeate flux but there is a challenge of homogeneous distribution in the membrane matrix and perpendicular alignment. Furthermore, CNTs are not cost-effective and are attributed to inducing toxicity after releasing in the environment, making them less encouraging for large-scale fabrication. Conversely, graphene-based materials are comparatively inexpensive alternatives and can provide better mechanical flexibility, and anti-biofouling features to membranes. Additionally, negative impact on environment and human health are less significant. CNFs also have a lower price, but their membranes provide much lower flux. MXene-based membranes have excellent anti-biofouling and separation features, but the cost is very high and needs to be substantially reduced. However, MXenes are newly introduced family of 2DNMs, which could be the potential candidate for next-generation membranes fabrication as they possess excellent hydrophilic and conductive properties. Nevertheless, many a challenge remains, but with research progress, 2DNMs are expected to show a promising influence on the next generation of nanocomposite membranes for various separation applications.

## 6. Conclusions

This review provides a state-of-the-art account on the synthesis and application of engineered 2DNMs for different types of membrane fabrications and its use for emerging water/wastewater purification technologies. The fundamental design strategies of 2DNMs membranes are discussed with an emphasis on their physicochemical properties, underlying mechanism, and targeted applications in different scenarios. Moreover, this review also aims to guide the design of next-generation 2DNMs-based membranes for the development of selective, multifunctional, programmable membranes for different types of water effluent treatment. A perspective on the pressing challenges and emerging opportunities in 2DNMs enabled membranes synthesis and application for wastewater treatment is also presented.

## Figures and Tables

**Table 1:** Synthesis procedure of different 2DNM membranes.

Type of Membrane	Synthesis Methods	References
PSF/Graphene oxide (GO)	For the preparation of membrane, casting solution was prepared by homogeneous dispersion of GO in PSF solution. The membrane was cast on an A4-sized non-woven polyester fabric for providing mechanical support.	[49]
PES/Polyaniline modified GO	Nanocomposite membranes using phase inversion method. PANI@GO nanoparticles were dispersed in DMAc as polymer solvent and then membrane was cast on a glass plate using a homemade applicator with 200 µm thickness and immersed immediately in DW as a non-solvent bath.	[50]
PVC/Carbon nanotubes (plasma treated)	In the first step, carbon nanotubes were functionalized with Ar/O_2_; then, plasma-functionalized CNTs were deposited onto the internal surface of the hollow-fiber membrane (PVC) with a specific CNT mass loading of 44.2 g m^−2^.	[51]
PVDF/Oxidized MCNTs + PAA	This membrane was fabricated by implanting CNTs into the pore channels of a ceramic (α-alumina) support by chemical vapor deposition method. The im- planted CNTs are oxidized with concentrated nitric acid at room temperature and chitosan is employed for filling intertube-CNT gaps.	[52]
PVDF/APTEs-HNTs	APTES-grafted HNTs were used on functionalized novel polyvinylidene fluoride (PVDF) nanofiltration membranes. Membrane was fabricated by traditional phase-inversion method.	[53]
PVA/CS+ aminated MCNTs	Amino-functionalized MWCNT-NH_2_ was prepared in first step then grafted onto membrane by phase-inversion method. PEG was utilized to improve pore capacity of CS/PVA.	[54]
GO/Torlon^®^composite	GO/Torlon^®^ composite membrane was fabricated by layer-by-layer method. Graphene oxide framework was constructed on Torlon 4000T-MV polymer in spin coating procedure.	[55]
MXene/PES composite	Multilayered Ti_3_C_2_T_x_ MXene was produced by etching of Ti_3_AlC_2_ with 49% HF and delaminated in DMSO. Then, the delaminated MXene solution was filtered through a PES UF membrane in a dead-end membrane set-up.	[56]
Self-crosslinked MXene membrane (SCMMs)	MXene membranes (SCMMs) were fabricated via the self-crosslinking reaction (−OH + −OH = −O− + H_2_O) between the neighboring MXene nanosheets by the facile thermal treatment of the pristine MXene membranes (PMMs).	[57]

**Table 2:** The available treatment technologies for heavy metals and their comparison with membrane filtration [15,23,86,110].

Treatment Technology	Advantages	Disadvantages
Precipitation	Simple process, low-cost method, use of cheap and accessible materials as precipitating agents	Higher number of chemical regents use, toxic secondary waste (sludge) generation, extra operational cost for secondary waste disposal or regenerative treatment
Coagulation/Flocculation		volume of final solid waste production, high operation coat
Membrane Filtration	High separation selectivity, lesser space requirement, low pressure required	High operation cot, slow selectivity, high energy consumption, and membrane fouling
Reverse Osmosis	Efficient method, able to bear fluctuating ion concentrations in the feed	Low selectivity and permeability, high pressure requirement (20–100 bars) makes it expensive, and membrane fouling
Electrochemical Methods	Good removal efficiency, higher contaminant selectivity, metal recovery with high purity	High operation rate because of membrane fouling and high energy consumption high energy consumption for both separation and electrode regeneration.
Ion Exchange	No secondary waste generation, time efficient,	Not all ion exchanger is suitable for metal removal, low selectivity, high treatment cost,
Adsorption	Low cost, high metal binding capacity	Low selectivity

**Table 3:** Removal of heavy metals by 2DNMs membranes.

Membrane/Adsorbent	Metal Ion	Concentration	Flux (L/m^2^/h/bar)	pH	Adsorption Capacity (mg/g)	Removal Rate	References
PSF/Graphene oxide (GO)	Pb^2+^	50 ppm	15.48	6.7	78.5	95	[49]
PES/Polyaniline modified GO	Pb^2+^	5 ppm	5.5	6	202	98	[50]
PTFE/PVA@GO	Cu^2+^	20 ppm	−	5.7	72.6	−	[109]
PVC/Carbon nanotubes (plasma treated)	Zn^2+^	500 ppb	44.4	5-9	−	>90	[51]
PVDF/Oxidized MCNTs + PAA	Ni^+^	20 ppm	40.08	7	5.306	53.12	[52]
PVDF/Functionalized halloysite nanotube	Cu^2+^	5 ppm	14.1	−	0.499	47.9	[111]
PVA/Cs+ aminated MCNTs	Cu^2+^	10 ppm	−	5	28.3	100	[54]
GO/Torlon^®^ composite	Pb, Ni, Zn	1000 ppm	4.7	5.34	−	95.88 99.74 98.07	[55]
MoS_2_ Nano sheets	Ag	20 ppm	−	6	4000	99%	[56]

## Data Availability

Not applicable.

## References

[1] FLahlou Z., Mackey H.R., Al-Ansari T. (2021). Wastewater reuse for livestock feed irrigation as a sustainable practice: A socio-environmental-economic review. J. Clean. Prod..

[2] Collivignarelli M.C., Abbà A., Miino M.C., Caccamo F.M., Torretta V., Rada E.C., Sorlini S. (2021). Disinfection of wastewater by uv-based treatment for reuse in a circular economy perspective. Where are we at?. Int. J. Environ. Res. Public Health.

[3] MPickett T., Roberson L.B., Calabria J.L., Bullard T.J., Turner G., Yeh D.H. (2020). Regenerative water purification for space applications: Needs, challenges, and technologies towards “closing the loop”. Life Sci. Space Res..

[4] Ang W.L., Mohammad A.W. (2020). State of the art and sustainability of natural coagulants in water and wastewater treatment. J. Clean. Prod..

[5] Aziz M. (2021). The Removal of Selected Inorganics from Municipal Membrane Bioreactor Wastewater Using UF/NF/RO Membranes for Water Reuse Application: A Pilot-Scale Study. Membranes.

[6] El-sayed M.E.A. (2020). Nanoadsorbents for water and wastewater remediation. Sci. Total Environ..

[7] Jaspal D., Malviya A. (2020). Composites for wastewater purification: A. review. Chemosphere.

[8] Ma X.C., Li X.K., Wang X.W., Liu G.G., Zuo J.L., Wang S.T., Wang K. (2020). Impact of salinity on anaerobic microbial community structure in high organic loading purified terephthalic acid wastewater treatment system. J. Hazard Mater..

[9] Homaeigohar S., Elbahri M. (2017). Graphene membranes for water desalination. NPG Asia Mater..

[10] Curcio E., di Profio G., Fontananova E., Drioli E. (2015). Membrane technologies for seawater desalination and brackish water treatment. Advances in Membrane Technologies for Water Treatment: Materials, Processes and Applications.

[11] Hussain A., Janson A., Matar J.M., Adham S. (2021). Membrane distillation: Recent technological developments and advancements in membrane materials. Emergent Mater..

[12] Li N., Lu X., He M., Duan X., Yan B., Chen G., Wang S. (2021). Catalytic membrane-based oxidation-filtration systems for organic wastewater purification: A review. J. Hazard. Mater..

[13] Moradihamedani P. (2021). Recent advances in dye removal from wastewater by membrane technology: A review. Polym. Bull..

[14] Tufa R.A., di Profio G., Fontananova E., Avci A.H., Curcio E. (2018). Forward osmosis, reverse electrodialysis and membrane distillation: New integration options in pretreatment and post-treatment membrane desalination process. Current Trends and Future Developments on (Bio-) Membranes: Membrane Desalination Systems: The Next Generation.

[15] Wenten I.G. (2016). Reverse osmosis applications: Prospect and challenges. Desalination.

[16] Silva M.A., Hilliou L., de Amorim M.T.P. (2020). Fabrication of pristine-multiwalled carbon nanotubes/ cellulose acetate composites for removal of methylene blue. Polym. Bull..

[17] MSilva A., Felgueiras H.P., de Amorim M.T.P. (2020). Carbon based membranes with modified properties: Thermal, morphological, mechanical and antimicrobial. Cellulose.

[18] Leaper S., Abdel-Karim A., Gorgojo P. (2021). The use of carbon nanomaterials in membrane distillation membranes: A review. Front. Chem. Sci. Eng..

[19] Li Z.K., Liu Y., Li L., Wei Y., Caro J., Wang H. (2019). Ultra-thin titanium carbide (MXene) sheet membranes for high-efficient oil/water emulsions separation. J. Membr. Sci..

[20] Ma W., Li Y., Gao S., Cui J., Qu Q., Wang Y., Huang C., Fu G. (2020). Self-Healing and Superwettable Nanofibrous Membranes with Excellent Stability toward Multifunctional Applications in Water Purification. ACS Appl. Mater. Interfaces.

[21] Shahzad A., Rasool K., Miran W., Nawaz M., Jang J., Mahmoud K.A., Lee D.S. (2017). Two-Dimensional Ti3C2Tx MXene Nanosheets for Efficient Copper Removal from Water. ACS Sustain. Chem. Eng..

[22] Yang Z., Gao D., Zhang J., Xu Q., Shi S., Tao K., Xue D. (2015). Realization of high Curie temperature ferromagnetism in atomically thin MoS2 and WS2 nanosheets with uniform and flower-like morphology. Nanoscale.

[23] Shahzad A., Miran W., Rasool K., Nawaz M., Jang J., Lim S.R., Lee D.S. (2017). Heavy metals removal by EDTA-functionalized chitosan graphene oxide nanocomposites. RSC Adv..

[24] Bai R.G., Muthoosamy K., Manickam S., Hilal-Alnaqbi A. (2019). Graphene-based 3D scaffolds in tissue engineering: Fabrication, applications, and future scope in liver tissue engineering. Int. J. Nanomed..

[25] Shahzad A., Rasool K., Nawaz M., Miran W., Jang J. (2018). Heterostructural TiO 2 / Ti 3 C 2 T x (MXene) for photocatalytic degradation of antiepileptic drug carbamazepine. Chem. Eng. J..

[26] Shahzad A., Rasool K., Miran W., Nawaz M., Jang J., Mahmoud K.A., Lee D.S. (2018). Mercuric ion capturing by recoverable titanium carbide magnetic nanocomposite. J. Hazard. Mater..

[27] Rao C.N.R., Maitra U., Waghmare U.V. (2014). Extraordinary attributes of 2-dimensional MoS2 nanosheets. Chem. Phys. Lett..

[28] Yang G., Zhang D., Wang C., Liu H., Qu L., Li H. (2019). A novel nanocomposite membrane combining bn nanosheets and go for effective removal of antibiotic in water. Nanomaterials.

[29] Le-Clech P. (2010). Membrane bioreactors and their uses in wastewater treatments. Appl. Microbiol. Biotechnol..

[30] Flemming H.C. (1997). Reverse osmosis membrane biofouling. Exp. Therm. Fluid Sci..

[31] Tijing L.D., Woo Y.C., Choi J.S., Lee S., Kim S.H., Shon H.K. (2015). Fouling and its control in membrane distillation-A review. J. Membr. Sci..

[32] Cheng H., Guan Q., Villalobos L.F., Peinemann K.-V., Pain A., Hong P.-Y. (2019). Understanding the antifouling mechanisms related to copper oxide and zinc oxide nanoparticles in anaerobic membrane bioreactors. Environ. Sci. Nano.

[33] Eshed M., Lellouche J., Matalon S., Gedanken A., Banin E. (2012). Sonochemical Coatings of ZnO and CuO Nanoparticles Inhibit Streptococcus mutans Biofilm Formation on Teeth Model. Langmuir.

[34] Tang C.Y., Chong T.H., Fane A.G. (2011). Colloidal interactions and fouling of NF and RO membranes: A review. Adv. Colloid Interface Sci..

[35] Djurišić A.B., Leung Y.H., Ng A.M.C., Xu X.Y., Lee P.K.H., Degger N., Wu R.S.S. (2015). Toxicity of Metal Oxide Nanoparticles: Mechanisms, Characterization, and Avoiding Experimental Artefacts. Small.

[36] Zhang R., Liu Y., He M., Su Y., Zhao X., Elimelech M., Jiang Z. (2016). Antifouling membranes for sustainable water purification: Strategies and mechanisms. Chem. Soc. Rev..

[37] Li H., Chen V. (2010). Membrane Fouling and Cleaning in Food and Bioprocessing. Membrane Technology.

[38] Chen W., Su Y., Peng J., Dong Y., Zhao X., Jiang Z. (2011). Engineering a robust, versatile amphiphilic membrane surface through forced surface segregation for ultralow flux-decline. Adv. Funct. Mater..

[39] Zeng M., Chen M., Huang D., Lei S., Zhang X., Wang L., Cheng Z. (2021). Engineered two-dimensional nanomaterials: An emerging paradigm for water purification and monitoring. Mater. Horiz..

[40] Cheng Y., Pu Y., Zhao D. (2020). Two-Dimensional Membranes: New Paradigms for High-Performance Separation Membranes. Chem. Asian J..

[41] Cheng L., Liu G., Zhao J., Jin W. (2021). Two-Dimensional-Material Membranes: Manipulating the Transport Pathway for Molecular Separation. Acc. Mater. Res..

[42] Nasir A.M., Goh P.S., Ismail A.F. (2019). Synthesis route for the fabrication of nanocomposite membranes. Nanocomposite Membranes for Water and Gas Separation.

[43] Yang Q., Mi B. (2013). Nanomaterials for Membrane Fouling Control: Accomplishments and Challenges. Adv. Chronic Kidney Dis..

[44] Liu H., Wang H., Zhang X. (2015). Facile Fabrication of Freestanding Ultrathin Reduced Graphene Oxide Membranes for Water Purification. Adv. Mater..

[45] Zhang Z., Yang S., Zhang P., Zhang J., Chen G., Feng X. (2019). Mechanically strong MXene/Kevlar nanofiber composite membranes as high-performance nanofluidic osmotic power generators. Nat. Commun..

[46] Ling Z., Ren C.E., Zhao M.Q., Yang J., Giammarco J.M., Qiu J., Barsoum M.W., Gogotsi Y. (2014). Flexible and conductive MXene films and nanocomposites with high capacitance. Proc. Natl. Acad. Sci. USA.

[47] .Ashori A., Hasanzadeh S. (2014). Removal of Acid Orange 7 from aqueous solution using magnetic graphene/chitosan: A promising nano-adsorbent. Int. J. Biol. Macromol..

[48] Mohedas A.H., Xing X., Armstrong K.A., Cuny G.D., Yu P.B. (2015). Sol-Gel Fabrication of a Non-Laminated Graphene Oxide Membrane for Oil/Water Separation. J. Mater. Chem. A.

[49] Mukherjee R., Bhunia P., De S. (2016). Impact of graphene oxide on removal of heavy metals using mixed matrix membrane. Chem. Eng. J..

[50] Ghaemi N., Zereshki S., Heidari S. (2017). Removal of lead ions from water using PES-based nanocomposite membrane incorporated with polyaniline modified GO nanoparticles: Performance optimization by central composite design. Process. Saf. Environ. Prot..

[51] Farid M.U., Luan H.Y., Wang Y., Huang H., An A.K., Khan R.J. (2017). Increased adsorption of aqueous zinc species by Ar/O2 plasma-treated carbon nanotubes immobilized in hollow-fiber ultrafiltration membrane. Chem. Eng. J..

[52] Tofighy M.A., Mohammadi T. (2014). Synthesis and characterization of ceramic/carbon nanotubes composite adsorptive membrane for copper ion removal from water. Korean J. Chem. Eng..

[53] Zeng G., He Y., Zhan Y., Zhang L., Pan Y., Zhang C., Yu Z. (2016). Novel polyvinylidene fluoride nanofiltration membrane blended with functionalized halloysite nanotubes for dye and heavy metal ions removal. J. Hazard. Mater..

[54] Salehi E., Madaeni S.S., Rajabi L., Derakhshan A.A., Daraei S., Vatanpour V. (2013). Static and dynamic adsorption of copper ions on chitosan/polyvinyl alcohol thin adsorptive membranes: Combined effect of polyethylene glycol and aminated multi-walled carbon nanotubes. Chem. Eng. J..

[55] Zhang Y., Zhang S., Gao J., Chung T.S. (2016). Layer-by-layer construction of graphene oxide (GO) framework composite membranes for highly efficient heavy metal removal. J. Membr. Sci..

[56] Wang Z., Sim A., Urban J.J., Mi B. (2018). Removal and Recovery of Heavy Metal Ions by Two-dimensional MoS2 Nanosheets: Performance and Mechanisms. Environ. Sci. Technol..

[57] Lu Z., Wei Y., Deng J., Ding L., Li Z.K., Wang H. (2019). Self-Crosslinked MXene (Ti3C2Tx) Membranes with Good Antiswelling Property for Monovalent Metal Ion Exclusion. ACS Nano.

[58] MAl-Ghouti A., Al-Kaabi M.A., Ashfaq M.Y., Da’na D.A. (2019). Produced water characteristics, treatment and reuse: A review. J. Water Process. Eng..

[59] Fakhru’l-Razi A., Pendashteh A., Abdullah L.C., Biak D.R.A., Madaeni S.S., Abidin Z.Z. (2009). Review of technologies for oil and gas produced water treatment. J. Hazard. Mater..

[60] Chakrabarty B., Ghoshal A.K., Purkait M.K. (2010). Ultrafiltration of oil-in-water emulsion: Analysis of fouling mechanism. Membr. Water Treat..

[61] Gupta R.K., Dunderdale G.J., England M.W., Hozumi A. (2017). Oil/water separation techniques: A review of recent progresses and future directions *J*. Mater. Chem. A.

[62] Alammar A., Park S.H., Williams C.J., Derby B., Szekely G. (2020). Oil-in-water separation with graphene-based nanocomposite membranes for produced water treatment. J. Membr. Sci..

[63] Humoud M.S., Roy S., Mitra S. (2020). Enhanced performance of carbon nanotube immobilized membrane for the treatment of high salinity produced water via direct contact membrane distillation. Membranes.

[64] Ao C., Yuan W., Zhao J., He X., Zhang X., Li Q., Xia T., Zhang W., Lu C. (2017). Superhydrophilic graphene oxide@electrospun cellulose nanofiber hybrid membrane for high-efficiency oil/water separation. Carbohydr. Polym..

[65] Kota A.K., Kwon G., Choi W., Mabry J.M., Tuteja A. (2012). Hygro-responsive membranes for effective oilg-water separation. Nat. Commun..

[66] Yu L., Han M., He F. (2017). A review of treating oily wastewater. Arab. J. Chem..

[67] Al-Anzi B.S., Siang O.C. (2017). Recent developments of carbon based nanomaterials and membranes for oily wastewater treatment. RSC Adv..

[68] Li X., Xu H., Liu J., Zhang J., Li J., Gui Z. (2016). Cyclonic state micro-bubble flotation column in oil-in-water emulsion separation. Sep. Purif. Technol..

[69] Noamani S., Niroomand S., Rastgar M., Sadrzadeh M. (2019). Carbon-based polymer nanocomposite membranes for oily wastewater treatment. NPJ Clean Water.

[70] Jayaramulu K., Datta K.K.R., Rösler C., Petr M., Otyepka M., Zboril R., Fischer R.A. (2016). Biomimetic superhydrophobic/superoleophilic highly fluorinated graphene oxide and ZIF-8 composites for oil-water separation. Angew. Chem. Int. Ed..

[71] Prince J.A., Bhuvana S., Anbharasi V., Ayyanar N., Boodhoo K.V.K., Singh G. (2016). Ultra-wetting graphene-based PES ultrafiltration membrane—A novel approach for successful oil-water separation. Water Res..

[72] Namasivayam M., Shapter J. (2017). Factors affecting carbon nanotube fillers towards enhancement of thermal conductivity in polymer nanocomposites: A review. J. Compos. Mater..

[73] Maphutha S., Moothi K., Meyyappan M., Iyuke S.E. (2013). A carbon nanotube-infused polysulfone membrane with polyvinyl alcohol layer for treating oil-containing waste water. Sci. Rep..

[74] Tai M.H., Gao P., Yong B., Tan L., Sun D.D., Leckie J.O. (2014). Highly Efficient and Flexible Electrospun Carbon-Silica Nano fi brous Membrane for Ultrafast Gravity-Driven Oil-Water Separation. ACS Appl. Mater. Interfaces.

[75] Liu H., Cao C.Y., Wei F.F., Huang P.P., Sun Y.B., Jiang L., Song W.G. (2014). Flexible macroporous carbon nanofiber film with high oil adsorption capacity. J. Mater. Chem. A.

[76] Pandey R.P., Rasool K., Rasheed P.A., Mahmoud K.A. (2018). Reductive Sequestration of Toxic Bromate from Drinking Water Using Lamellar Two-Dimensional Ti3C2TX (MXene). ACS Sustain. Chem. Eng..

[77] Rasool K., Helal M., Ali A., Ren C.E., Gogotsi Y. (2016). Antibacterial Activity of Ti3C2Tx MXene. ACS Nano.

[78] Rasool K., Pandey R.P., Rasheed P.A., Buczek S., Gogotsi Y., Mahmoud K.A. (2019). Water treatment and environmental remediation applications of two-dimensional metal carbides (MXenes). Mater. Today.

[79] Saththasivam J., Wang K., Yiming W., Liu Z., Mahmoud K.A. (2019). A flexible Ti3C2Tx (MXene)/paper membrane for efficient oil/water separation. RSC Adv..

[80] Membrane Technologies for Seawater Desalination and Brackish Water Treatment. https://www.sciencedirect.com/science/article/pii/B9781782421214000137.

[81] Abraham J., Vasu K.S., Williams C.D., Gopinadhan K., Su Y., Cherian C.T., Dix J., Prestat E., Haigh S.J., Grigorieva I.V. (2017). Tunable sieving of ions using graphene oxide membranes. Nat. Nanotechnol..

[82] You Y., Sahajwalla V., Yoshimura M., Joshi R.K. (2016). Graphene and graphene oxide for desalination. Nanoscale.

[83] Zheng Z., Grünker R., Feng X. (2016). Synthetic Two-Dimensional Materials: A New Paradigm of Membranes for Ultimate Separation. Adv. Mater..

[84] Epsztein R., DuChanois R.M., Ritt C.L., Noy A., Elimelech M. (2020). Towards single-species selectivity of membranes with subnanometre pores. Nat. Nanotechnol..

[85] Seo D.H., Pineda S., Woo Y.C., Xie M., Murdock A.T., Ang E.Y.M., Jiao Y., Park M.J., Lim S.I., Lawn M. (2018). Anti-fouling graphene-based membranes for effective water desalination. Nat. Commun..

[86] Surwade S.P., Smirnov S.N., Vlassiouk I.V., Unocic R.R., Veith G.M., Dai S., Mahurin S.M. (2015). Water desalination using nanoporous single-layer graphene. Nat. Nanotechnol..

[87] Yang Y., Yang X., Liang L., Gao Y., Cheng H., Li X., Zou M., Ma R., Yuan Q., Duan X. (2019). Large-area graphene-nanomesh/carbon-nanotube hybrid membranes for ionic and molecular nanofiltration. Science.

[88] Kang Y., Xia Y., Wang H., Zhang X. (2019). 2D Laminar Membranes for Selective Water and Ion Transport. Adv. Funct. Mater..

[89] Ihsanullah (2019). Carbon nanotube membranes for water purification: Developments, challenges, and prospects for the future. Sep. Purif. Technol..

[90] Jia Y.X., Li H.L., Wang M., Wu L.Y., Hu Y.D. (2010). Carbon nanotube: Possible candidate for forward osmosis. Sep. Purif. Technol..

[91] An A.K., Lee E.J., Guo J., Jeong S., Lee J.G., Ghaffour N. (2017). Enhanced vapor transport in membrane distillation via functionalized carbon nanotubes anchored into electrospun nanofibers. Sci. Rep..

[92] Bhadra M., Roy S., Mitra S. (2016). Flux enhancement in direct contact membrane distillation by implementing carbon nanotube immobilized PTFE membrane. Sep. Purif. Technol..

[93] Sun Y., Li S., Zhuang Y., Liu G., Xing W., Jing W. (2019). Adjustable interlayer spacing of ultrathin MXene-derived membranes for ion rejection. J. Membr. Sci..

[94] Momodu M.A., Anyakora C.A. (2010). Heavy Metal Contamination of Ground Water: The Surulere Case Study. Res. J. Environ. Earth Sci..

[95] Kosuri S. (2016). Study of Polylysine and Chitosan Nanoparticles Synthesized Using Various Cross-Linkers and Their Applications for Heavy Metal Ion Recovery.

[96] Fu F., Wang Q. (2011). Removal of heavy metal ions from wastewaters: A review. J. Environ. Manag..

[97] Namasivayam C., Kadirvelu K. (1999). Uptake of mercury (II) from wastewater by activated carbon from an unwanted agricultural solid by-product: Coirpith. Carbon.

[98] Lu X., Huangfu X., Ma J. (2014). Removal of trace mercury(II) from aqueous solution by in situ formed Mn-Fe (hydr)oxides. J. Hazard. Mater..

[99] Cui L., Wang Y., Gao L., Hu L., Yan L., Wei Q., Du B. (2015). EDTA functionalized magnetic graphene oxide for removal of Pb(II), Hg(II) and Cu(II) in water treatment: Adsorption mechanism and separation property. Chem. Eng. J..

[100] Jung M.C. (2008). Contamination by Cd, Cu, Pb, and Zn in mine wastes from abandoned metal mines classified as mineralization types in Korea. Environ. Geochem. Health.

[101] Karimi R.F. (2011–2012). A study of the Heavy Metal Extraction Process Using Emulsion Liquid Membranes. Master’s Thesis.

[102] Muhammad S., Shah M.T., Khan S. (2011). Health risk assessment of heavy metals and their source apportionment in drinking water of Kohistan region, northern Pakistan. Microchem. J..

[103] (2013). United Nations Environment Programme. Global Mercury Assessment.

[104] Aydın H., Bulut Y., Yerlikya C. (2008). Removal of copper (II) from aqueous solution by adsorption onto low-cost adsorbents. J. Environ. Manag..

[105] Aslam M., Rais S., Alam M., Pugazhendi A. (2013). Adsorption of Hg (II) from aqueous solution using adulsa (*Justicia adhatoda*) leaves powder: Kinetic and equilibrium studies. J. Chem..

[106] Wang Y., Ye G., Chen H., Hu X., Niu Z., Ma S. (2015). Functionalized metal-organic framework as a new platform for efficient and selective removal of cadmium (II) from aqueous solution. J. Mater. Chem. A.

[107] AKommu, Namsani S., Singh J.K. (2016). Removal of heavy metal ions using functionalized graphene membranes: A molecular dynamics study. RSC Adv..

[108] Zunita M., Makertiharta I.G.B.N., Irawanti R., Prasetya N., Wenten I.G. (2018). Graphene Oxide-Inorganic Composite Membrane: A Review. IOP Conference Series: Materials Science and Engineering.

[109] Zhang Y., Zhang S., Chung T.S. (2015). Nanometric Graphene Oxide Framework Membranes with Enhanced Heavy Metal Removal via Nanofiltration. Environ. Sci. Technol..

[110] Hodkin D.J., Stewart D.I., Graham J.T., Burke I.T. (2016). Coprecipitation of 14C and Sr with carbonate precipitation: The importance of reaction kinetics and recrystallization pathways. Sci. Total. Environ..

[111] Huang Z.Q., Cheng Z.F. (2020). Recent advances in adsorptive membranes for removal of harmful cations. J. Appl. Polym. Sci..

[112] Patel M., Kumar R., Kishor K., Mlsna T., Pittman C.U., Mohan D. (2019). Pharmaceuticals of Emerging Concern in Aquatic Systems: Chemistry, Occurrence, Effects, and Removal Methods. Chem. Rev..

[113] Soni R., Pal A.K., Tripathi P., Lal J.A., Kesari K., Tripathi V. (2020). An overview of nanoscale materials on the removal of wastewater contaminants. Appl. Water Sci..

[114] MPervez N., Balakrishnan M., Hasan S.W., Choo K.-H., Zhao Y., Cai Y., Zarra T., Belgiorno V., Naddeo V. (2020). A critical review on nanomaterials membrane bioreactor (NMs-MBR) for wastewater treatment. NPJ Clean Water.

[115] Han Y., Xu Z., Gao C. (2013). Ultrathin graphene nanofiltration membrane for water purification. Adv. Funct. Mater..

[116] Ding L., Wei Y., Wang Y., Chen H., Caro J., Wang H. (2017). A Two-Dimensional Lamellar Membrane: MXene Nanosheet Stacks, *Angew*. Chem. Int. Ed..

[117] Sun L., Ying Y., Huang H., Song Z., Mao Y., Xu Z., Peng X. (2014). Ultrafast molecule separation through layered WS2 nanosheet membranes. ACS Nano.

[118] Ang H., Hong L. (2017). Polycationic Polymer-Regulated Assembling of 2D MOF Nanosheets for High-Performance Nanofiltration. ACS Appl. Mater. Interfaces.

[119] Liu P., Hou J., Zhang Y., Li L., Lu X., Tang Z. (2020). Two-dimensional material membranes for critical separations. Inorg. Chem. Front..

[120] Ozgun H., Dereli R.K., Ersahin M.E., Kinaci C., Spanjers H., van Lier J.B. (2013). A review of anaerobic membrane bioreactors for municipal wastewater treatment: Integration options, limitations and expectations. Sep. Purif. Technol..

[121] Fathizadeh M., Xu W.L., Shen M., Jeng E., Zhou F., Dong Q., Behera D., Song Z., Wang L., Shakouri A. (2019). Antifouling UV-treated GO/PES hollow fiber membranes in a membrane bioreactor (MBR). Environ. Sci. Water Res. Technol..

[122] Neoh C.H., Noor Z.Z., Mutamim N.S.A., Lim C.K. (2016). Green technology in wastewater treatment technologies: Integration of membrane bioreactor with various wastewater treatment systems. Chem. Eng. J..

[123] Goswami L., Kumar R.V., Borah S.N., Manikandan N.A., Pakshirajan K., Pugazhenthi G. (2018). Membrane bioreactor and integrated membrane bioreactor systems for micropollutant removal from wastewater: A review. J. Water Process. Eng..

[124] Zhou T., Ma L., Gan M., Wang H., Hao C. (2019). Sandwich-structured hybrids: A facile electrostatic self-assembly of exfoliated titania nanosheets and polyaniline nanoparticles and its high visible-light photocatalytic performance. J. Phys. Chem. Solids.

[125] Xiao G., Wang Y., Xu S., Li P., Yang C., Jin Y., Sun Q., Su H. (2019). Superior adsorption performance of graphitic carbon nitride nanosheets for both cationic and anionic heavy metals from wastewater. Chin. J. Chem. Eng..

[126] Kashefi S., Borghei S.M., Mahmoodi N.M. (2019). Covalently immobilized laccase onto graphene oxide nanosheets: Preparation, characterization, and biodegradation of azo dyes in colored wastewater. J. Mol. Liq..

[127] Ghalamchi L., Aber S., Vatanpour V., Kian M. (2019). A novel antibacterial mixed matrixed PES membrane fabricated from embedding aminated Ag3PO4/g-C3N4 nanocomposite for use in the membrane bioreactor. J. Ind. Eng. Chem..

